# Extrapyramidal syndromes of chronic kidney disease and dialysis (diabetic uremic syndrome) with reversible parkinsonism and lentiform fork sign: A case report and literature review including metformin-induced encephalopathy

**DOI:** 10.1016/j.heliyon.2023.e14255

**Published:** 2023-03-03

**Authors:** Takeo Sakurai, Hiroshi Nishida

**Affiliations:** Department of Neurology, Gifu Prefectural General Medical Center, Gifu 500-8717, Japan

**Keywords:** Extrapyramidal syndromes of chronic kidney disease and dialysis, Diabetic uremic syndrome, Metformin-induced encephalopathy, Parkinsonism, Lentiform fork sign, Metformin

## Abstract

Diabetic uremic syndrome has been rarely reported in patients on maintenance dialysis for diabetic nephropathy who present subacutely with neurological symptoms and bilateral basal ganglia lesions. There are also a few reports on metformin-induced encephalopathy, which is clinically similar to diabetic uremic syndrome. Because some patients with each of these diseases also have metabolic acidosis, it is speculated that these two diseases may have the same pathology. Recently, the term “extrapyramidal syndromes of chronic kidney disease and dialysis” (EPS-CKDD), with associated diagnostic criteria, has been proposed to describe these conditions, and metformin use is considered a risk factor for developing these syndromes. We report a case of a patient on maintenance hemodialysis for diabetic nephropathy who was taking metformin and developed subacute parkinsonism and bilateral basal ganglia lesions that rapidly improved after discontinuation of metformin with continued maintenance hemodialysis. We should ascertain whether patients with EPS-CKDD are taking metformin because it may be inappropriately prescribed for end-stage renal disease. If metformin has been prescribed, it should be discontinued immediately; its discontinuation may lead to rapid symptom recovery and improved prognosis.

## 1 Introduction

Diabetic uremic syndrome has been rarely reported in patients on maintenance dialysis for diabetic nephropathy who present subacutely with neurological symptoms and bilateral basal ganglia lesions. Wang et al. [1] were the first to propose this syndrome, and subsequent reports on the syndrome have been scattered. Parkinsonism is the most common neurological symptom, with other symptoms including impaired consciousness, chorea, and dyskinesia [2]. Metabolic acidosis is considered the main possible cause [1,3], and therefore the syndrome has also been rarely reported in patients with chronic renal disease without diabetes mellitus and patients who are not on maintenance dialysis [4,5]. Other patients were reported to have metformin-induced encephalopathy [[6], [7], [8], [9], [10], [11], [12]], with similar characteristics for patient background, clinical presentation, and imaging findings to patients with diabetic uremic syndrome. The exact mechanism of metformin-induced encephalopathy is unclear; it is thought to result from an overdosage, a failure to extract the treatment, an individual idiosyncratic susceptibility, or possibly the accumulation of multiple metformin doses in the brain [10]. Recently, Manickavasagar et al. [13] proposed the name “extrapyramidal syndromes of chronic kidney disease and dialysis” (EPS-CKDD) and diagnostic criteria for these syndromes, and the risk factors listed for these conditions include metformin use, dialysis, diabetes, female sex, and thiamine deficiency. These syndromes may represent a single continuum of disease, with inconsistent terminology and a lack of diagnostic criteria, making it difficult to identify them as such. We present the case of a patient on maintenance dialysis for diabetic nephropathy who was taking metformin and presented subacutely with parkinsonism and bilateral basal ganglia lesions that showed rapid improvement after discontinuation of metformin with continued maintenance dialysis. We also discuss the related literature, including cases of metformin-induced encephalopathy.

## 2 Case report

**Fig. 1:**
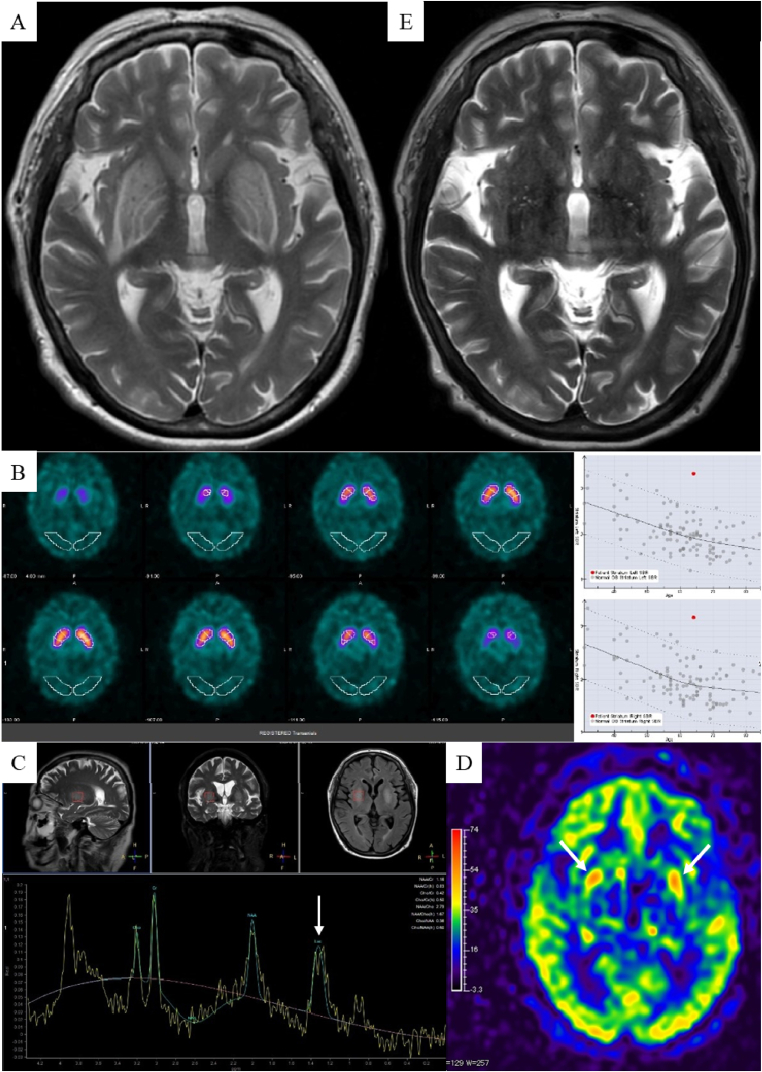
Brain imaging findings for the patient. (A) T2-weighted brain magnetic resonance imaging (MRI) revealed bilateral basal ganglia lesions with the lentiform fork sign. (B) A dopamine transporter scan showed no decreased accumulation. (C) Magnetic resonance spectroscopy demonstrated a lactate peak at 1.3 ppm (arrow). (D) Arterial spin labeling showed hyperperfusion in the lesions (arrows). (E) T2-weighted brain MRI performed about one month after admission showed that the bilateral basal ganglia lesions were disappearing.

The patient was a 64-year-old woman who was diagnosed with diabetes mellitus 5 years previously and had been on maintenance dialysis for diabetic nephropathy for the last 2 years. Her medical history included hypertension and breast cancer. She was treated for diabetes mellitus with metformin, sitagliptin, and insulin. She had been taking metformin for at least 2 years. She denied any alcohol consumption, and was taking no vitamins. In the middle of the month prior to presenting, she experienced vomiting and diarrhea that resolved after approximately 1 week. Early in the month she presented, she slowly developed difficulty in moving her extremities, a low-volume voice, and slow speech. Her symptoms progressed gradually, leading to difficulty in getting up or walking steadily. She was hospitalized on the 22nd day of that month. She did not exhibit any anorexia on admission. Her blood pressure was high at 163/76 mmHg. Neurological examination showed no disturbance of consciousness, mask-like face, hypophonia, bradykinesia, rigidity of both upper limbs, left terminal tremor, weakness of proximal muscles of both lower limbs, gait disturbance (short and shuffling steps, reduced arm swing), and positive Babinski and Chaddock signs in the left lower extremity, which indicated parkinsonism and pyramidal tract signs. A blood test showed no abnormalities; specifically, vitamin B1, thyroid function, and lactate levels were normal. A spinal fluid test showed no abnormalities. Blood gas analysis was normal without acidosis. T2-weighted brain magnetic resonance imaging (MRI) showed high-intensity areas in the bilateral lenticular nucleus with the lentiform fork sign (Fig. 1A). Diffusion-weighted imaging showed equal intensity, and a dopamine transporter scan revealed no decreased accumulation, but rather increased levels in the lesions (Fig. 1B). Magnetic resonance spectroscopy (MRS) demonstrated a lactate peak (Fig. 1C), and arterial spin labeling (ASL) showed hyperperfusion in the lesions (Fig. 1D). After admission, maintenance dialysis was continued under the same conditions as before and metformin was discontinued. Her symptoms had almost completely resolved after approximately 2 weeks. About 1 month after admission, the basal ganglia lesions observed on T2-weighted brain MRI had completely disappeared (Fig. 1E).

## 3 Discussion

Our patient's clinical presentation was consistent with diabetic uremic syndrome, as proposed by Wang et al. [1], because of her background of receiving maintenance dialysis for diabetic nephropathy and having subacute onset of parkinsonism and bilateral basal ganglia lesions. In addition, our patient met the diagnostic criteria proposed by Manickavasagar et al. [13], so we diagnosed her as having EPS-CKDD. An effective treatment for this syndrome has not yet been clarified, as some cases resolved spontaneously with continued maintenance dialysis, while others presented with sequelae. Our case showed rapid improvement of her symptoms and MRI findings despite only discontinuation of metformin with continued maintenance dialysis. There have been scattered reports of the syndrome since it was first proposed by Wang et al. [1]. Meanwhile, cases of metformin-induced encephalopathy have been reported. There have been eight reported cases of metformin-induced encephalopathy with bilateral basal ganglia lesions [[6], [7], [8], [9], [10], [11], [12]], all of which involved diabetes mellitus and end-stage renal disease with bilateral basal ganglia lesions including the lentiform fork sign, and thus the clinical pictures are almost identical to those of diabetic uremic syndrome (Table 1). However, in these cases, the symptoms appeared during metformin use and promptly improved after its discontinuation, suggesting that metformin was strongly involved. There are no established diagnostic criteria for metformin-induced encephalopathy, which is diagnosed on the basis of MRI findings and improvement after discontinuation of metformin, and those findings also seem to apply to the present case. In the first report on diabetic uremic syndrome by Wang et al. [1], most cases were accompanied by metabolic acidosis. Furthermore, in the report by Kumar et al. [3], almost all cases of certain diseases, including methanol intoxication and mitochondrial diseases, presenting with the lentiform fork sign were accompanied by metabolic acidosis, suggesting that metabolic acidosis may be the main cause of this condition. Both diabetic uremic syndrome and metformin-induced encephalopathy were accompanied by metabolic acidosis in some cases, and thus the two diseases may have a common underlying pathology. The reason for our case showing no acidosis on admission is speculated as follows. Our patient may have developed acidosis after taking metformin that was corrected by maintenance dialysis, and the maintenance dialysis may also have partially eliminated the metformin [11]. However, maintenance dialysis only was insufficient, because the blood levels of metformin remained high and metformin tends to accumulate in several tissues including the brain [14], and thus metformin should be discontinued. Another possible mechanism is a direct effect of metformin on the central nervous system [15]. Our case supports the proposed diagnostic criteria for EPS-CKDD, which includes diabetic uremic syndrome and metformin encephalopathy, and confirms that metformin use is a risk factor.

**Table 1:** Background characteristics, coexisting medical problems, and clinical outcomes of patients reported to have metformin-induced encephalopathy with basal ganglia lesions.

Case	Reference	Age and sex	Past history	Duration of dialysis	Duration of metformin use	Duration from symptom onset	Neurological symptoms	Acidosis	Prognosis
1	Berrada et al. [6]	49 F	DM, ESRD	ND	5 days	Rapid	Parkinsonism, DOC	Absent	A clinical and radiological outcome was favorable 1 month later.
2	Simon ＆ Thomas [7]	63 M	DM, ESRD, old cerebrovascular accident	2 years	Long-term	1 month	Parkinsonism, DOC, mild weakness, asterixis	Present	Improvement in sensorium after a few days. Brain MRI showed resolution of changes after 2 months.
3	McGarvey et al. [8]	44 F	DM, ESRD, HT, obesity, hypothyroidism	3 months	Several months (increased dose 7 weeks before)	4 weeks	Parkinsonism	ND	Recovered over the next few days without neurological sequelae.
4	Fernandes et al. [9]	63 F	DM, ESRD	ND	1 week	Subacute	Parkinsonism	ND	ND
5	Kang et al. [10]	62 M	DM, ESRD, HT	5 years	5 weeks	10 days	DOC, dysarthria, gait disturbance, myoclonus	ND	Neurological abnormalities had almost completely resolved after three regular hemodialysis sessions. MRI revealed complete resolution.
6	Kang et al. [10]	55 F	DM, ESRD	4 years	4 weeks	3 days	Dysarthria, myoclonus, involuntary movement of legs	ND	Neurological abnormalities had almost completely resolved after three hemodialysis sessions. MRI after 2 weeks revealed complete resolution.
7	Jung et al. [11]	51 M	DM, ESRD	2 years	3 months	1 month	Dysarthria, mild hemiparesis, myoclonus, asterixis, trismus	ND	Symptoms and signs had completely disappeared at 7 days after admission. MRI after 3 weeks showed a marked improvement.
8	Mewborne et al. [12]	55 M	DM, ESRD, HT	ND	18 months	2 days	DOC, slurred speech, generalized weakness	Present	Full recovery was observed.

DOC, disturbance of consciousness; DM, diabetes mellitus; ESRD, end-stage renal disease; HT, hypertension; ND, not documented.

In previous reports, metformin-induced encephalopathy showed a good prognosis with rapid improvement of symptoms and MRI findings in all cases after discontinuation of metformin [[6], [7], [8], [9], [10], [11], [12]]. Meanwhile, the prognosis of diabetic uremic syndrome is generally not good, with some patients presenting with sequelae and others dying from complications. There are seven reported cases of patients with diabetic uremic syndrome who were taking metformin [5,[16], [17], [18], [19], [20], [21]], and these patients had a worse prognosis than those with metformin-induced encephalopathy because they required a longer time to exhibit symptom reduction and improved MRI findings, or they had presented with sequelae. However, there is a possibility of publication bias, and thus the results cannot be simply compared. In fact, immediate cessation of metformin did not result in recovery in all cases, as demonstrated by the 20 cases of EPS-CKDD reported by Manickavasagar et al. [13], of which 19 were on metformin at the time of onset. When metformin was discontinued (or, in one case, in the absence of metformin treatment at the time of onset), nine patients recovered, nine continued to experience residual symptoms, and two died.

Based on the above observations, we should ascertain whether patients with EPS-CKDD (diabetic uremic syndrome) are taking metformin. If metformin is being taken, it should be discontinued immediately. Currently, metformin is contraindicated when the estimated glomerular filtration rate (eGFR) is < 30 ml/min/1.73 m^2^ because of the risk of lactic acidosis, but it may be inappropriately prescribed.

Regarding MRS, the present case demonstrated a lactate peak in the lesions, similar to previously reported cases of diabetic uremic syndrome and metformin-induced encephalopathy [8,22]. Regarding blood flow in the lesions, the present case showed increases on ASL. In diabetic uremic syndrome, there are reports showing increased [19] or decreased [22,23] blood flow based on single-photon emission computed tomography, computed tomographic perfusion, and ASL. We speculate that these findings reflect whether the lesions are suffering from irreversible damage depending on the degree of vasogenic edema or demyelination. For metformin-induced encephalopathy, there are no reports on the blood flow in the lesions.

## 4 Conclusion

Diabetic uremic syndrome and metformin-induced encephalopathy are clinically similar and may have a common underlying pathology, such as metabolic acidosis. We should ascertain whether patients with EPS-CKDD (diabetic uremic syndrome) are taking metformin because it may be inappropriately prescribed for end-stage renal disease. If they are, it should be discontinued immediately, and this discontinuation may lead to rapid symptom recovery and improved prognosis.

## Production notes

### Author contribution statement

All authors listed have significantly contributed to the investigation, development and writing of this article.

### Funding statement

This research did not receive any specific grant from funding agencies in the public, commercial, or not-for-profit sectors.

### Data availability statement

Data included in article/supplementary material/referenced in article.

### Declaration of interest’s statement

The authors declare no conflict of interest.
